# Original quantitative research - A prospective study of financial worry, mental health changes and the moderating effect of social support among Canadian adolescents during the COVID-19 pandemic

**DOI:** 10.24095/hpcdp.44.3.04

**Published:** 2024-03

**Authors:** Jessica A. Goddard, Valerie F. Pagnotta, Markus J. Duncan, Matthew Sudiyono, William Pickett, Scott T. Leatherdale, Karen A. Patte

**Affiliations:** 1 Department of Health Sciences, Brock University, St. Catharines, Ontario, Canada; 2 Department of Public Health Sciences, Queen’s University, Kingston, Ontario, Canada; 3 School of Public Health Sciences, University of Waterloo, Waterloo, Ontario, Canada

**Keywords:** financial worry, adolescents, COVID-19 pandemic, Canada, anxiety, depression, social support

## Abstract

**Introduction:**

The COVID-19 pandemic intensified the impact of risk factors for adolescent mental health, including financial worry. Social support has shown to protect from negative mental health during times of stress. We examined the effect of financial worry on changes in anxiety and depression symptoms among Canadian adolescents prior to and during the pandemic, and assessed whether social support from family and friends moderated any changes.

**Methods:**

We analyzed 2-year linked data from the 2018/19 (pre-pandemic) and 2020/21 (during-pandemic) waves of the COMPASS study, with reports from 12995 Canadian secondary school students. A series of multilevel linear regressions were conducted to examine the main hypotheses under study.

**Results:**

Students scored an average (SD) of 7.2 (5.8) on the anxiety (GAD-7) and 10.0 (6.5) on the depression (CESD-10) scales; 16.1% reported they experienced financial worry during the pandemic. Financial worry was a strong and significant predictor of increased anxiety scores (+1.7 score between those reporting “true/mostly true” versus “false/mostly false”) during the pandemic, but not for depression scores. Low family and friend support were associated with anxiety, and low family support was associated with depression. No significant interactions were detected between social support and financial worry.

**Conclusion:**

Pandemic-related financial worry was significantly associated with anxiety in our large sample of Canadian adolescents. Clinical and public health initiatives should be aware of adolescents’ financial worry and its associations with anxiety during times of crisis.

**Highlights**

- The COVID-19 pandemic intensified risk factors to adolescent mental health.
- Pandemic-related financial worry was significantly associated with changes in anxiety scores, but not depression scores.
- Low family and friend support were associated with anxiety, and low family support was associated with depression; however, no significant interactions were identified with financial worry.
- Public health policy-makers, clinicians and parents should be aware that financial worry is associated with anxiety among adolescents and may be exacerbated by economic crises.
- Family support programs that focus on promoting adolescent mental health, given the association between parental financial worry and their children’s mental health, may be a suggested next step.

## Introduction

Adolescent mental health continued to be a global priority during the COVID-19 pandemic.^1^ The pandemic impacted the lives of adolescents across the world, with school closures and shifts to online education, physical distancing and social isolation, and the closure of social and recreational facilities.^2^ These pandemic-related measures are believed to have contributed to worsening mental health among adolescents^2^ and widened inequities in access to support.^3^jva For instance, a large cross-sectional study found that up to 70% of Canadian youth experienced a deterioration in mental health, despite that more than one-third had no prior indication of mental health concerns.^4^ Prior to the pandemic, approximately 1 in 4 Canadian youth experienced a mental disorder.^5^ Given that the first onset of adult mental disorders usually occurs during childhood and adolescence, it is important to address risk factors early in the life course.^6^

A potential risk factor for mental disorders in adolescents that, for some, intensified during the pandemic is financial stress.^7^ Financial stress (or financial worry) is defined as the uncertainty, concern and fear of income instability, unemployment and debt.^8^,^9^ The mechanism by which financial worry and adolescent mental health are linked is complex and driven through indirect and direct pathways.^10^,^11^ According to the family stress model, stress experienced by parents because of financial hardships may result in disrupted parenting (e.g. inconsistent and harsh parenting, reduced family time and withdrawing support), which increases the risk of their children experiencing poor mental health.^12^ Effects of parental financial stress on the child vary depending on the child’s developmental age, with adolescents particularly at risk for adverse mental health outcomes because of their greater awareness and understanding of financial issues and potential pressures to contribute to family finances.^13^,^14^

Over the past 20 years, Canadian household saving rates have declined, with many families lacking financial assets to fulfill basic needs in the event of an emergency.^15^ The onset of the pandemic in March 2020, and the subsequent economic downturn, led to the emergence and exacerbation of financial uncertainties for many families. During the early pandemic phases, 17% of Canadian adults expressed major concern about their ability to uphold financial commitments and meet their basic needs.^16^ Racialized people and those with low socioeconomic status were disproportionately affected (e.g. higher rates of disease leading to loss of workdays, lower-paying jobs eliminated because of unsafe conditions/inability to work remotely).^17^,^18^ In response to these pandemic-related economic ramifications, the Canadian government introduced relief programs (e.g. tax rebates, income replacement) that financially supplemented 68.4% of Canadians.^19^

An important protective factor to adverse mental health outcomes among adolescents is social support. Social support, defined as interactions with individuals (e.g. family, friends) who provide physical and emotional support, has been linked with increased resilience, self-esteem, well-being and life satisfaction among adolescents.^20^,^21^ Social support has been shown to protect against depression and anxiety.^21^ The importance of social relationships, particularly during periods of stress and crises (e.g. economic downturns), is well documented.^22^ A recent systematic review included survey studies conducted during the pandemic that affirmed this association; having social and familial support was associated with having better mental health outcomes.^23^ A recent study has shown social support to be a coping mechanism protecting against adverse mental health stemming from financial stress.^24^

There are few published studies on the relationship between financial worry and adolescent mental health in the context of the pandemic. While one US study identified financial stress as a predictor of depression among adolescents,^25^ most research has focussed on adults.^26^,^27^ Evidence exists for the positive effects of social support on adolescent mental health during the pandemic,^23^ and one US study identified a moderating effect of parental support on adolescents’ financial stress and mental health.^28^ However, to our knowledge, no studies have examined financial worry and mental health, and the moderating role of social support, among Canadian adolescents.

To address these gaps, we developed a prospective study design using pre- and during-pandemic data from a large sample of Canadian adolescents. We examined the relationship between financial worry and changes in anxiety and depression from before to during the pandemic and assessed the potential moderating role of family and friend support. The findings may inform the prevention of mental disorders by (1) identifying an underrecognized risk factor at a critical developmental stage, and (2) exploring social support as a protective factor and target for interventions.

## Methods

***Design and participants***

We used two-year linked student-level data from two waves of the Cannabis, Obesity, Mental health, Physical activity, Alcohol, Smoking, and Sedentary behaviour (COMPASS) Study spanning the 2018/19 (pre-pandemic) and 2020/21 (during-pandemic) waves. COMPASS is an ongoing prospective study that collects annual survey data from a rolling cohort of students in Grades 9 to 12 (Secondary I–V in Quebec) across secondary schools in four Canadian provinces (British Columbia, Alberta, Ontario and Quebec).^29^

Students are recruited using an active-information passive-consent protocol. Student-level data were collected using the COMPASS student questionnaire.^29^ The 2018/19 questionnaire was completed during class time via paper-and-pencil surveys; the 2020/21 questionnaire was completed during class time in person or, because of pandemic-related school closures, at home through an emailed link to an online survey.^30^ Data from 2019/20 were not used because data collection interruptions occurred halfway through the year with school closures due to the first COVID-19 lockdown.

This study used linked-longitudinal data from 12995 participants from 111 secondary schools. In the 2018/19 and 2020/21 survey years, average response rates across schools were 84.2% and 58.0%, respectively. Primary reasons for non-linkage and nonparticipation included students graduating out of the cohort, missing data on linkage items, and collection methods (e.g. student absences, scheduling data collection outside of class time).

Additional details about COMPASS study methods can be found online (https://uwaterloo.ca/compass-system/) or in print.^29^ COMPASS study data are available upon request by completing a COMPASS Data Usage Application at https://uwaterloo.ca/compass-system/information-researchers/data-usage-application. The datasets used in the current study are available from the corresponding author on reasonable request.

***Ethics approval***

All procedures received ethics approval from the University of Waterloo (ORE#30118), Brock University (REB#18-099), Centre intgr universitaire de sant et des services sociaux du Centre-Ouest-de-l’ile-de-Montral (CIUSSS) de la Capitale-Nationale–Universit Laval (#MP-13-2017-1264) and participating school boards, including the use of active-information passive-consent parental permission protocols.

***Consent to participate***

To be considered eligible using the active-information passive-consent protocol, schools informed parents/guardians about the study, and after a reasonable time had passed (e.g. 2 weeks) parents/guardians had to have informed the COMPASS school representative that they did not want their child to participate in the study. In addition, students had to assent to participate and were able to withdraw their assent to participate at any time.

***Mental health measures***

**Depression**

Symptoms of depression were measured using the 10-item Center for Epidemiologic Studies Depression Scale (CESD-10), which comprises questions related to sadness, loneliness and loss of interest during the past 7 days.^31^ CESD-10 scores are calculated by summing individual item response scores. Scores range from 0 to 30, and those greater than or equal to 10 indicate clinically relevant depression symptomology.

This tool has been validated for use in adolescent populations against the original CESD-20.^32^ In the present study, the internal consistency was .82 (good) and .84 (good) for the 2018/19 and 2020/21 survey years, respectively.

**Anxiety**

Symptoms of anxiety were measured using the Generalized Anxiety Disorder 7-item Scale (GAD-7).^33^ This tool uses self-reported questions related to nervousness, excessive or uncontrollable worrying, irritability, and restlessness over a 2-week period.^33^ GAD-7 scores are calculated by summing individual item response scores. Scores range from 0 to 21, and those greater than or equal to 10 indicate clinically relevant anxiety symptomology.

This scale has been validated to determine anxiety symptoms and clinical anxiety in adolescent populations.^34^ In this study, the internal consistency was .89 (good) and .91 (excellent) for the 2018/19 and 2020/21 survey years, respectively.

**Financial worry**

Financial worry was measured through the single item “I am worried about my family being able to pay bills and expenses” that was included in the 2020/21 survey COVID-19 module (i.e. “How true are the following statements about COVID-19 for you right now?”). Response options used a 5-point Likert scale. Responses were condensed to three categories because of small cell sizes: true/mostly true; neutral/don’t know; and mostly false/false. This measure was adapted from the COVID-19 Adolescent Symptom and Psychological Experience Questionnaire^35^ and pretested by the COMPASS study team.

***Social support***

We selected, a priori, the following theory-driven variables as potential effect modifiers: family support (“I can talk about my problems with my family”) and friend support (“I can talk about my problems with my friends”). Both items were in the 2018/19 and 2020/21 COMPASS surveys and both were derived from the Multidimensional Scale of Perceived Social Support (MSPSS).^36^ Response options on a 5-point Likert scale were condensed into the following three categories because of small cell sizes: strongly agree/agree; neither agree nor disagree; and disagree/strongly disagree.

***Covariates***

The following covariates described in the 2020/21 survey were included in analyses: age (years); gender (male, female, I describe my gender in a different way/prefer not to say [responses collapsed because of small cell sizes]); race/ethnicity (White, Black, Asian, Latin American/Hispanic, Other/Mixed); province (Ontario, Alberta, British Columbia, Quebec), as experiences may have varied between locations; and school learning mode (in person, online or hybrid [alternating online and in-person school days]), as there may have been differences in pandemic-related experiences, depending on the school setting.

***Statistical analysis***

We conducted univariate analysis to collect participants’ basic characteristics. To account for missing data, multiple imputation was conducted for all variables using the *mice* (multivariate imputation by chained equation) and *miceadds* packages in RStudio version 4.2.0 (PBC, Boston, MA, US).^37^ Individual-level missing values were imputed using predictive means matching. Items from the CESD-10 and GAD-7 were imputed prior to scoring to reduce bias.^38^ Missing data on the CESD-10 and GAD-7 were 8.5% and 7.9%, respectively, and as a result, 10 imputations were sufficient according to guidelines established by Graham et al.;^39^ 50 iterations were deemed appropriate after visually examining convergence via trace plots.

To assess school clustering, intraclass correlation coefficients (ICC) were calculated via addition of a random intercept (school-level) to an unadjusted regression model for both anxiety and depression. The ICC values for both models were within the acceptable range of less than 3% (0.5% for both depression and anxiety); as a result, school clustering did not account for enough variation in outcomes to require its inclusion in subsequent models.^40^

We conducted a series of multilevel linear regressions to examine the association between financial worry and changes in mental health scores, and whether this association was moderated by family or friend support. To assess change, timepoint 2 for depression and anxiety were regressed on their respective baseline (time 1) score, in addition to adjusting for covariates. Models from the multiple imputed datasets were pooled using Rubin’s rules. Hypothesis testing was conducted by comparing a series of iterative nested models based on the Akaike information criterion (AIC) and analysis of variance (ANOVA) using the D2 pooled likelihood ratio tests to determine if adding predictor variables of interest improved upon the nested reduced model.

**Figure 1:**
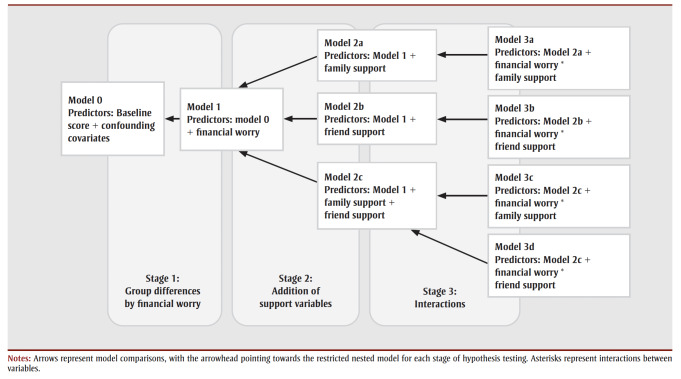
A diagram of nested models and comparisons

For both depression and anxiety score changes, the models were compared in three iterative stages: (1) whether adding financial worry improved upon a reduced model of confounding covariates alone; (2) whether the addition of family and/or friend support improved on the full model from Stage 1; and (3) whether interactions between family and/or friend support and financial worry improved upon the full models identified in Stage 2 (illustrated in Figure 1). Pairwise post-hoc tests were conducted to evaluate group comparisons on statistically significant models; *p* values were adjusted using the Bonferroni method.

We also conducted tests of interaction effects between financial worry and gender in models 2a, 2b and 2c (not reported); however, these results showed no significant gender interactions and nonsignificant model improvement compared to the reduced models and so were not included in further analyses.

## Results

***Participant characteristics***

The mean (SD) age of the 12 995 participants at time 2 (during the COVID-19 pandemic in 2020/21) was 15.9 (1.1) years, with the majority identifying as female (55.3%), White (80.2%), attending a secondary school in Quebec (62.8%) and reporting a hybrid school learning mode during the pandemic (53.4%).

A total of 16.1% of participants reported experiencing financial worry during the pandemic. The mean (SD) GAD-7 score was 7.2 (5.8) and the mean (SD) CESD-10 score was 10.0 (6.5). The proportions of participants reporting clinically relevant GAD-7 and CESD-10 scores were 30.2% and 46.0%, respectively. More than half of the sample agreed that they were able to talk to about their problems with their family (56.6%), and nearly three-quarters agreed that they could talk about their problems to their friends (73.9%) (Table 1).

**Table 1:** Characteristics of 2020/21 sample (n = 12 995)

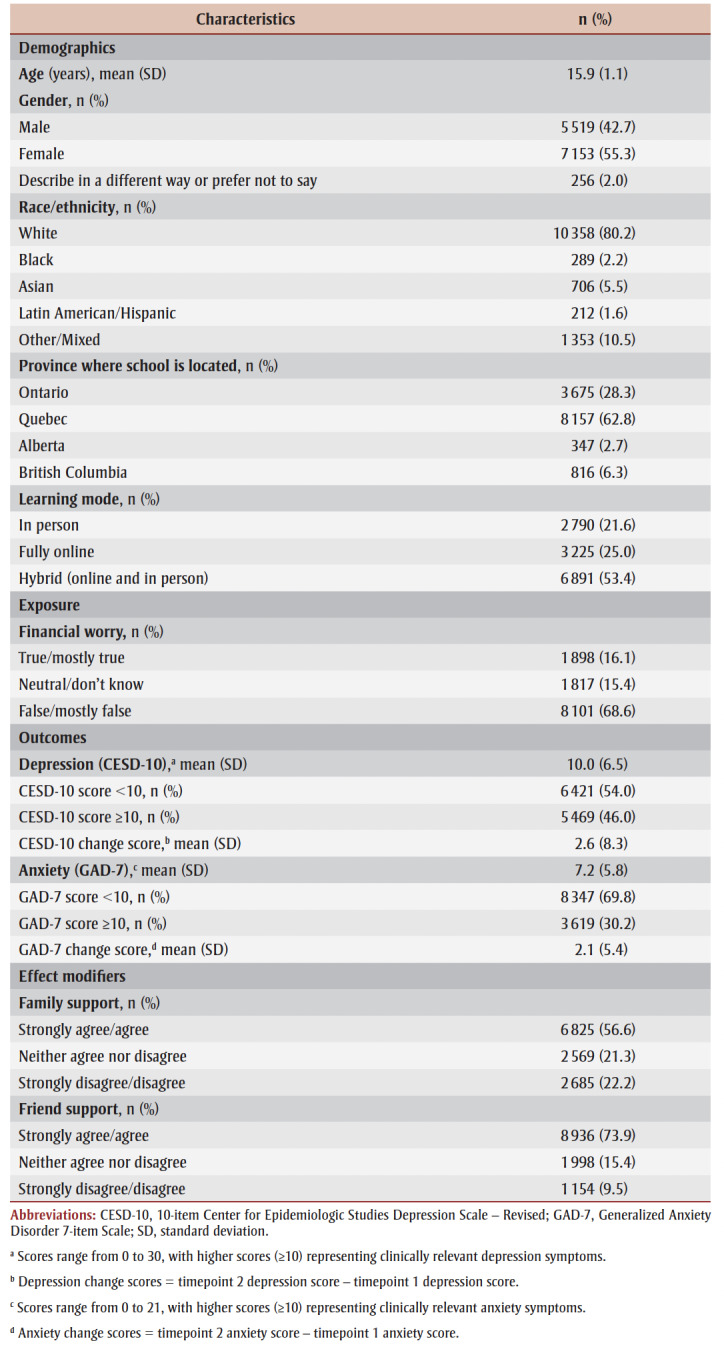

***Model comparison statistics and likelihood ratio tests for mental health changes***

For anxiety, the addition of financial worry (Model 1) was a significant improvement on the null model (Model 0), exhibiting a lower AIC (mean [SD], AIC=77325.8 [50.0]). Using Model 1 as the new reduced model, the inclusion of family and friend support, both individually (Models 2a and 2b) and collectively (Model 2c), showed a significant improvement. Model 2c emerged as the best fitting model with lowest AIC (76518.2 [46.9]) relative to Model 1 (Table 2).

**Table 2:** Main effect model comparison fit indices and likelihood ratio test summary for depression and anxiety change

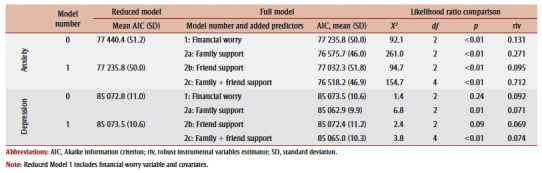

For depression, the inclusion of financial worry (Model 1) did not significantly improve upon Model 0. When evaluating the inclusion of support relative to Model1, family support alone (Model 2a), and coupled with friend support (Model 2c), showed significant model improvement. Model 2a (including family support) was identified as having best model fit with lowest AIC (85062.9 [9.9]).

***Post-hoc comparisons using the Bonferroni method for financial worry***

Differences in average anxiety scores followed a positive trend; after adjusting for covariates, those who reported having financial worry had the highest estimated marginal mean anxiety score at timepoint 2 (9.0), followed by those reporting “neutral/don’t know” (8.0) and then “false/mostly false” (7.3). In turn, the largest differences in anxiety change scores were exhibited between those who reported financial worry, relative to those without (+1.7); differences in anxiety scores between each category were significant (*p*<0.01).

For depression, no significant differences between groups were identified (Table 3).

**Table 3:** Post-hoc test summary of group comparisons for anxiety and depression

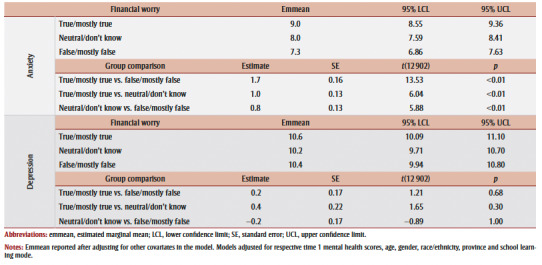

***Post-hoc comparisons for social support***

For anxiety, significant differences were shown between family support responses. Those without family support had the highest estimated marginal mean anxiety score at time 2 relative to those with family support (9.7 versus 7.2). Friend support showed a similar difference in marginal mean anxiety scores (8.9 versus 7.9).

For depression, significant differences were found in the estimated marginal mean depression score among those without family support compared to those with such support (10.7 versus 10.2) (Table 4).

**Table 4:** Post-hoc test summary of group comparisons for anxiety and depression for best fitting models from Stage 2

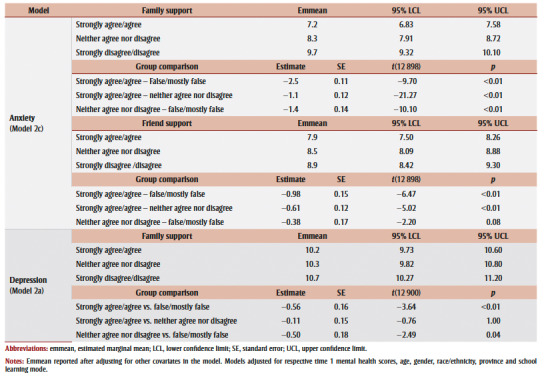

***Interaction between financial worry and social support***

No meaningful or statistically significant interactions were detected for both anxiety and depression (Table 5). Models that further adjusted for the reciprocal support measure (that was not included in the interaction term) similarly showed nonsignificant results.

**Table 5:** Model comparisons at Stage 3: Testing family and friend support interactions

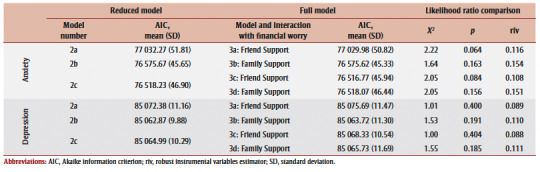

## Discussion

We examined the effect of financial worry on changes in anxiety and depression from prior to and during the pandemic among Canadian adolescents and whether social support moderated this relationship. Our most important findings were: (1) financial worry was significantly associated with changes in anxiety from pre- to during-pandemic timepoints, but not with changes in depression; (2) having low family and friend support was associated with changes in anxiety scores, but only low family support (and friend support when coupled with family support) was associated with changes in depression; and (3) there were no significant interactions between financial worry and family and friend support with regard to changes in adolescent anxiety and depression.

On average, participants experienced an increase in both anxiety and depression scores during the pandemic relative to their pre-pandemic scores. This finding aligns with contemporary evidence. One systematic review of 21 studies (longitudinal and repeated cross-sectional designs) assessing mental health changes pre- and during the pandemic among youth (aged 0 to 24 years) identified increased depression and anxiety reported across most of studies included in their review.^1^ A global meta-analysis found the pooled prevalence of elevated depression and anxiety disorders was 25.2% and 20.5%, respectively.^41^ The authors compared these prevalence estimates to pre-pandemic estimates and noted that adolescents struggling with a mental disorder approximately doubled during the pandemic.^41^

It is important to consider the context and clinical relevance of these mental health changes. From a developmental perspective, we expect to see an increase in anxiety and depression symptoms as adolescents age.^42^ Therefore, we cannot distinguish between developmental and pandemic-related effects on mental health in our study. To determine clinical relevance, 4-point and 6-point change scores on the GAD-7 have been suggested according to the minimal clinically important difference^43^ and Reliable Change Index,^44^ respectively. However, these changes apply to clinical samples with existing psychopathology and were developed to evaluate treatments to decrease patient scores to under the 10-point threshold.

The mean change scores in our study may not be clinically significant, but nevertheless may be relevant at a population level; a small increase may be sufficient for many individuals to reach or surpass the clinical threshold of 10. The only such guideline for the CESD-10 is the threshold score of 10 to determine clinically relevant symptomatology.^31^ Notably, our sample had a mean score of 10 on the CESD-10 during the pandemic, with nearly half of participants scoring above this threshold.

Financial worry was associated with heightened anxiety during the pandemic, but not with depression in our sample. Although anxiety and depression are highly co-morbid and share many similarities, these results are not necessarily surprising as anxiety is characterized by “worry” (e.g. nervousness, fear of the future) while amotivation and anhedonia are primary characteristics of depression.^45^ Some evidence suggests that anxiety disorders generally precede the presentation of depression.^46^ Thus, future research with longer follow-up is warranted.

In contrast to our findings, two US studies found relationships between youths’ stress about their family’s finances and depression^25^ and negative affect (e.g. sad, anxious, depressed)^28^ among adolescents.

The protective effects of social support on adolescent mental health are well established. Social support has been shown to mitigate adverse mental health outcomes and promote coping during periods of heightened stress and uncertainty, including during the COVID-19 pandemic.^23^,^28^ Our study reaffirmed these findings, identifying significant associations between both family and friend support with anxiety change scores, and family support, both individually and when paired with friend support, was associated with changes in depression. Our finding that family and friend support remained an important asset to the mental health of Canadian adolescents was encouraging. Many adolescents may have faced barriers in accessing support during the pandemic (e.g. restrictions to in-person socializing, school and facility closures).

No significant interactions resulted when examining the family and friend support as moderators of the relationships between pandemic-related financial worry and anxiety and depression. In contrast, a daily diary study conducted in the USA during the early pandemic identified a moderating effect of parental support on the relationship between financial uncertainty and negative affect among adolescents.^28^ Conflicting results may reflect differences across studies in timing, contexts or designs; a micro-longitudinal design was employed that did not account for participants’ pre-pandemic mental health. Research conducted prior to the pandemic is also inconsistent and has largely focussed on adult samples. For instance, slund et al^47^ identified social support as having a “buffering” role in adverse mental well-being attributable to financial stress among Swedish adults, whereas Viseu et al^24^ failed to identify a moderating effect of support on financial threat and stress, anxiety and depression among Portuguese adults during an economic crisis. Further research is warranted to confirm the tested moderating effects among Canadian adolescents.

While financial protections were implemented by the Canadian government, 16.1% of adolescents were worried about their family’s ability to pay bills as a result of the COVID-19 pandemic; this worry was associated with an increase in anxiety from their pre-pandemic levels. Public health policy-makers, clinicians and parents should be aware that financial worry is associated with anxiety among adolescents, which may be exacerbated by economic downturns, and that ensuring equitable access to mental health support is crucial to the early intervention of mental disorders.

Our study identified that social support remained relevant in the context of the pandemic and should continue to be a focus of mental health initiatives. However, our results suggest social support is not sufficient to significantly moderate the association between financial worry and mental health outcomes. In accordance with the family stress model,^12^ targeted family support programs may be effective in promoting mental health among adolescents,^48^ given the downstream effects of financial stress from parent to child.

***Strengths and limitations***

Important strengths of this study include the use of prospective data from a large sample of Canadian adolescents; robust statistical analyses that accounted for pre-pandemic mental health scores; and well-validated mental health scales.^32^,^34^ Our focus on adolescents was novel, given the limited evidence on mental health relative to financial stress in this demographic, particularly during the pandemic.

Limitations of the study also warrant comment. Selection bias may have been introduced, with the lower response rate (58%) during the pandemic. However, the survey was administered to students across the four largest Canadian provinces, including areas that differed in urbanicity and income, and private and public schools. While self-reported questionnaires are subject to potential recall error and social desirability bias, the passive-consent and confidentiality protocols may promote generalizability and honest reporting.^49^

The measures of financial worry and social support are single-item variables derived from scales that have been previously used and validated in adolescent populations.^35^,^36^ While single-item measures typically correlate with multi-item measures, they can have limitations when capturing more complex constructs.

The student questionnaire was designed to be brief for data quality and feasibility in a large school-based population-level study covering multiple domains. No measures of absolute financial status of adolescents’ families were available, given the difficulties that adolescents may have measuring household income. We were also unable to test for potential interaction effects by race/ethnicity, given the small cell sizes in some groups. The change from paper-and-pencil to online questionnaires during the pandemic may have impacted responses. Finally, baseline financial worry responses were not available as this measure was introduced in the pandemic questionnaire.

## Conclusion

Pandemic-related financial worry was significantly associated with anxiety in Canadian adolescents. While family and friend support are important protective factors for anxiety symptoms, and family support on depression symptoms, during the COVID-19 pandemic, they did not moderate the relationship between financial worry and mental health changes. Future research should continue to monitor the association between financial worry and adolescent mental health and explore and test other potential protective factors that may mitigate adverse effects.

## Acknowledgements

The authors would like to thank the schools, school boards and students that have participated in the COMPASS study and all COMPASS team members, staff, partners and youth engagement committee members. It takes a large team, many collaborators and particularly, students and schools themselves, to make this study possible.

## Funding

The COMPASS study has been supported by a bridge grant from the Canadian Institutes of Health Research (CIHR) Institute of Nutrition, Metabolism and Diabetes (INMD) through the “Obesity – Interventions to Prevent or Treat” priority funding awards (OOP-110788) awarded to STL; an operating grant from the CIHR Institute of Population and Public Health (IPPH) (MOP-114875) awarded to STL; a CIHR project grant (PJT-148562) awarded to STL; a CIHR bridge grant (PJT-149092) awarded to KAP/STL; a CIHR project grant (PJT-159693) awarded to KAP; a research funding arrangement with Health Canada (#1617-HQ-000012) awarded to STL; a CIHR-Canadian Centre on Substance Abuse (CCSA) team grant (OF7 B1-PCPEGT 410-10-9633) awarded to SL; and a project grant from the CIHR Institute of Population and Public Health (IPPH) (PJT-180262) awarded to STL and KAP.

A SickKids Foundation New Investigator Grant, in partnership with CIHR Institute of Human Development, Child and Youth Health (IHDCYH) (Grant No. NI21-1193), awarded to KAP, funds a mixed methods study examining the impact of the COVID-19 pandemic on youth mental health, leveraging COMPASS study data. A CIHR operating grant funds analysis of COVID-19 impacts on health behaviours in COMPASS (UIP 178846 CIHR; awarded to KAP). The COMPASS-Qubec project also benefits from funding from the Ministre de la sant et des services sociaux of the province of Quebec and the Direction rgionale de sant publique du CIUSSS de la Capitale-Nationale. KAP is the Canada Research Chair in Child Health Equity and Inclusion.

The funding sources had no role in the study design; in the collection, analysis and interpretation of data; in the writing of the manuscript; or in the decision to submit the article for publication.

## Conflicts of interest

Scott Leatherdale is one of this journal’s associate scientific editors, but has recused himself from the review process for this article.

The authors have no other conflicts of interest to declare.

## Authors’ contributions and statement

JAG: Conceptualization, formal analysis, writing – original draft, writing – review & editing.

VFP: Conceptualization, formal analysis, writing – original draft, writing – review & editing.

MS: Conceptualization, writing – review & editing.

KAP: Conceptualization, funding acquisition, supervision, writing – review & editing.

WP: Conceptualization, supervision, writing – review & editing.

STL: Conceptualization, funding acquisition, methodology, supervision, writing – review & editing.

MJD: Formal analysis, writing – review & editing.

All authors approved the final manuscript.

The content and views expressed in this article are those of the authors and do not necessarily reflect those of the Government of Canada.
